# Magnetic resonance imaging and neuropsychological findings for predicting of cognitive deterioration in memory clinic patients

**DOI:** 10.3389/fnagi.2023.1155122

**Published:** 2023-08-03

**Authors:** Kana Matsuda, Masaki Shinohara, Yuichiro Ii, Ken-ichi Tabei, Yukito Ueda, Naoko Nakamura, Yoshinori Hirata, Hidehiro Ishikawa, Hirofumi Matsuyama, Keita Matsuura, Masayuki Satoh, Masayuki Maeda, Ryo Momosaki, Hidekazu Tomimoto, Akihiro Shindo

**Affiliations:** ^1^Department of Dementia Prevention and Therapeutics, Mie University Graduate School of Medicine, Tsu, Japan; ^2^Department of Neurology, Mie University Graduate School of Medicine, Tsu, Japan; ^3^Department of Neuroradiology, Mie University Graduate School of Medicine, Tsu, Japan; ^4^Department of Rehabilitation Medicine, Mie University Graduate School of Medicine, Tsu, Japan

**Keywords:** mild cognitive impairment, mild dementia, hypertensive arteriopathy-small vessel disease score, cerebral amyloid angiopathy-small vessel disease score, modified cerebral amyloid angiopathy-small vessel disease score, memory clinic

## Abstract

**Objective:**

The severity of cerebral small vessel disease (SVD) on magnetic resonance imaging (MRI) has been assessed using hypertensive arteriopathy SVD and cerebral amyloid angiopathy (CAA)-SVD scores. In addition, we reported the modified CAA-SVD score including cortical microinfarcts and posterior dominant white matter hyperintensity. Each SVD score has been associated with cognitive function, but the longitudinal changes remain unclear. Therefore, this study prospectively examined the prognostic value of each SVD score, imaging findings of cerebral SVD, and neuropsychological assessment.

**Methods:**

This study included 29 patients diagnosed with mild cognitive impairment or mild dementia at memory clinic in our hospital, who underwent clinical dementia rating (CDR) and brain MRI (3D-fluid attenuated inversion recovery, 3D-double inversion recovery, and susceptibility-weighted imaging) at baseline and 1 year later. Each SVD score and neuropsychological tests including the Mini-Mental State Examination, Japanese Raven’s Colored Progressive Matrices, Trail Making Test -A/-B, and the Rivermead Behavioral Memory Test were evaluated at baseline and 1 year later.

**Results:**

Twenty patients had unchanged CDR (group A), while nine patients had worsened CDR (group B) after 1 year. At baseline, there was no significant difference in each SVD score; after 1 year, group B had significantly increased CAA-SVD and modified CAA-SVD scores. Group B also showed a significantly higher number of lobar microbleeds than group A at baseline. Furthermore, group B had significantly longer Japanese Raven’s Colored Progressive Matrices and Trail Making test-A times at baseline. After 1 year, group B had significantly lower Mini-Mental State Examination, Japanese Raven’s Colored Progressive Matrices, and Rivermead Behavioral Memory Test scores and significantly fewer word fluency (letters).

**Conclusion:**

Patients with worsened CDR 1 year after had a higher number of lobar microbleeds and prolonged psychomotor speed at baseline. These findings may become predictors of cognitive deterioration in patients who visit memory clinics.

## 1. Introduction

The incidence of dementia has declined over the decades in the United States and England (Matthews et al., 2016); however, the increase of the aging population continues to increase the number of patients with dementia. Dementia is one of the main social and public health problems worldwide. Alzheimer’s disease (AD) and vascular dementia are two major causes of dementia and are linked to cerebral small vessel disease (SVD) (Shindo et al., 2020).

The two major types of SVD are hypertensive arteriopathy (HA) and cerebral amyloid angiopathy (CAA). Magnetic resonance imaging (MRI) technology has made it possible to visualize lesions caused by SVD (Pantoni, 2010). HA and CAA share common MRI features of SVD, including white matter hyperintensities (WMH), enlargement of perivascular spaces (PVS), and cerebral microbleeds (MBs). However, the location and distribution of these radiological findings are different. In CAA, the anteroposterior distribution of WMH is posterior-dominant (Sekh et al., 2014). The enlargement of PVS in the basal ganglia (BG-PVS) is associated with hypertension, while patients with CAA show centrum semiovale PVS (CSO-PVS) (Charidimou et al., 2014). MBs in the BG, thalamus, or brainstem (i.e., deep MBs) indicate HA, while MBs in the lobar brain compartment are associated with CAA (Greenberg et al., 2009). Moreover, lacunar infarcts are associated with hypertension, and cortical superficial siderosis (cSS) is one of the MRI biomarkers in CAA (Linn et al., 2010). Cortical microinfarcts (CMIs) are small foci restricted to the cerebral cortex and caused by different pathological backgrounds, such as CAA, arteriosclerosis, and microembolism (Kövari et al., 2017). CMI has become detectable by MRI in clinical settings (Ii et al., 2019). Furthermore, neuroradiological findings obtained using 3T MRI might distinguish CMIs due to CAA and microembolisms (Ishikawa et al., 2020).

These SVD imaging features have been associated with cognitive decline (Staals et al., 2015). WMH is an independent risk factor for progression from mild cognitive impairment (MCI) to AD (Defrancesco et al., 2013), and several reports have shown that WMH is an aggravating factor for cognitive decline in AD (Brickman et al., 2008; de Leeuw et al., 2006; Park et al., 2011). WMH could also become the predictor for cognitive decline (Clancy et al., 2022). In addition, an increase of lobar MBs are associated with cognitive decline (Gregoire et al., 2013; Poels et al., 2012), and the number of lobar MBs become the predictor for cognitive decline (Gyanwali et al., 2021). Imaging markers for ischemic and hemorrhagic stroke, as well as SVD markers, can predict dementia in patients with stroke (Rost et al., 2022; Taniguchi et al., 2022), there are a few reports that SVD markers can be a predictor of cognitive decline in non-stroke patients prospectively.

In contrast, neuropsychological assessments also can predict cognitive decline; studies on MCI have reported that deterioration of verbal memory, executive functions or visual memory predict conversion to AD (Belleville et al., 2017) and deficits in verbal memory and psychomotor speed/executive function abilities strongly predicted conversion to AD (Tabert et al., 2006). Moreover, studies on aMCI (amnestic MCI) have reported that the rate of change in TMT-B over 1 year is a predictor of conversion to AD (Rozzini et al., 2007), and that memory tests can predict cognitive decline better than executive functions, attention, and working memory (Vyhnalek et al., 2022).

Recently, three types of MRI-based assessment scores have been developed for SVD, including HA-SVD, CAA-SVD, and modified CAA-SVD score (Klarenbeek et al., 2013; Staals et al., 2014; Charidimou et al., 2016; Matsuda et al., 2021). Each SVD score has been associated with cognitive function, but changes in each score and each item of the score longitudinal changes remain unclear. Therefore, this study aimed to examine prospectively the prognostic value for the deterioration of cognitive function with each SVD score, by comparing imaging findings of SVD and neuropsychological assessments in the patients of our memory clinic.

## 2. Materials and methods

### 2.1. Patients

**FIGURE 1:**
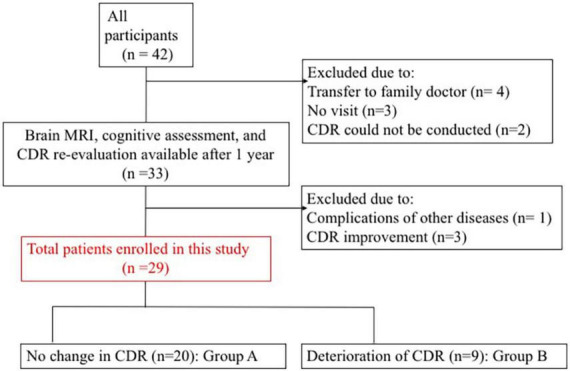
Patient enrolment process. CDR, clinical dementia rating; MRI, magnetic resonance imaging.

We prospectively registered 42 patients who consulted memory clinic in our hospital (Matsuda et al., 2021). Thirty-three of 42 patients were available for MRI, neuropsychological test, and CDR follow-up at 1 year. After excluding four patients with complications of other diseases or CDR improvement, total twenty-nine patients were enrolled in this study (Figure 1). Inclusion and exclusion criteria are shown in Supplementary Table 1.

Among the participants, 20 had no change in CDR (group A) and nine had CDR deterioration (group B). Table 1 presents the clinical characteristics at baseline evaluation. Groups A and B did not differ significantly in clinical characteristics. All procedures followed the clinical study guidelines of the Ethics Committee of Mie University Hospital and were approved by the internal review board (Registration number: 1596). A complete description of all procedures was provided to the patients, and written informed consent was obtained directly from patients or their caregivers. All patients were examined by neurologists with sufficient experience in examining patients with dementia.

**TABLE 1:** Participant characteristics.

Demographics		Group A, *N* = 20	Group B, *N* = 9	*p*
Age, years, mean (SD)		71.8 (2.4)	75.3 (2.1)	0.59
Education, years, mean (SD)		12.3 (0.5)	12.0 (0)	0.69
Duration of disease, months, mean (SD)		15.8 (4.9)	15.2 (5.6)	0.95
CDR-SB, score, mean (SD)		3.1 (0.3)	4.3 (0.7)	0.11
MCI (CDR 0.5), N (%)		15 (75)	6 (66.6)	0.64
Mild dementia (CDR 1), N (%)		5 (25)	3 (33.3)	0.64
modified Boston criteria (ver 1.5)	Probable CAA, N (%)	4 (20)	4 (44.4)	0.40
	Possible CAA, N (%)	8 (40)	2 (22.2)	0.26
Hypertension, N (%)		9 (45)	5 (55.5)	0.38
Dyslipidemia, N (%)		5 (25)	1 (11.1)	0.40

MCI, mild cognitive impairement; CDR-SB, clinical dementia rating sum of boxes; CAA, cerebral amyloid angiopathy; SD, standard deviation.

A diagnosis of MCI was made by CDR, and CDR 0.5 was diagnosed as MCI (Hughes et al., 1982). MCI was classified into an amnestic type (aMCI) or non-amnestic type (naMCI) depending on the presence or absence of memory impairment, respectively (Petersen, 2011). We diagnosed AD according to the National Institute on Aging–Alzheimer’s Association guidelines (McKhann et al., 2011). Vascular dementia was diagnosed according to the criteria set forth by the National Institute of Neurological Disorders and the Stroke Association International pour la Recherche et l’Enseignement en Neurosciences (NINDS-AIREN) (Román et al., 1993).

### 2.2. Neuropsychological assessments

The Mini-Mental State Examination (MMSE) (Mori et al., 1985) and Japanese Raven’s Colored Progressive Matrices (RCPM) (Raven, 1962) were used to quantify intellectual function. Memory was evaluated using the Rivermead Behavioral Memory Test (RBMT). The scores included a standard profile score (SPS) and screening score (SS) (Wilson et al., 1989). Constructional ability was assessed using the Mie Constructional Apraxia Scale (Satoh et al., 2016). Frontal lobe function was assessed using two tasks: Word Fluency (WF) and Trail-Making Test (TMT) -A/-B (Abe et al., 2004). The WF test consisted of category and letter domains. In the category WF task, participants were asked to name as many animals as possible in 1 min. In the letter WF task, participants were asked to name as many objects as possible in 1 min, beginning with each of the following four phonemes: *ka, sa, ta*, and *te*. The average scores for these four phonemes were used for statistical analyses.

Two speech therapists performed CDR, and the results were evaluated through a discussion between two neurologists and three speech therapists based on the CDR determination rules (Hughes et al., 1982).

### 2.3. MRI protocol

The MRI protocol was the same reported by Ii et al. (2019). Briefly, MRI studies were performed with a 3T MRI unit (Ingenia, Philips Medical System, Best, Netherlands) using an 8- or 32-channel phased-array head coil. We used T1- and T2-weighted and 3D fluid attenuated inversion recovery (FLAIR) images to evaluate WMH, lacunar infarcts, and PVS. Susceptibility-weighted image (SWI) sequences were used for the detection of MBs and cSS. CMIs were detected using 3D double inversion recovery (DIR) and 3D FLAIR. Axial double inversion recovery imaging was performed using two different inversion pulses. The long and short inversion times were defined as the intervals between the 180° inversion pulse and the 90° excitation pulse, respectively, which had been optimized for human brain imaging and were provided by the manufacturer.

Details of the 3D FLAIR: field of view 250 mm, matrix 256 × 184, section thickness 0.57 mm with over contiguous slices, TR/TE 6,000 ms/390 ms (shortest), TI 2,000 ms, number of signals acquired 2; and acquisition time 4 min 42 s; 3D DIR: field of view 250 mm, matrix 208 × 163, section thickness 0.65 mm with over contiguous slices, turbo spin echo factor 173, TR/TE 5,500 ms/298 ms; long inversion time (TI)/short TI 2,550 ms/450 ms, number of signals acquired 2; and acquisition time 5 min 13 s; SWI: field of view 230 mm, matrix 384 × 300, section thickness 1.0 mm with over contiguous slices, TR/TE 31 ms/7.2 ms, echo spacing 6.2 ms; number of echoes 4; number of signals acquired 1; flap angle 17°, and acquisition time 4 min 52 s; T1-weighted imaging (T1WI): field of view 260 mm, matrix 288 × 288, section thickness 0.9 mm, repetition time (TR)/echo time (TE) 7.6 ms (shortest)/3.6 ms (shortest), flip angle 10°, and acquisition time 4 min 42 s; T2-weighted imaging: field of view 220 mm, matrix 384 × 345, section thickness 3.0 mm, gap 0.5 mm, TR/TE 7,000 ms (shortest)/90 ms; and acquisition time 2 min 43 s (Table 2).

**TABLE 2:** MRI protocol.

	Plane	TR/TE (ms)	FA (°)	Matrix	Thickness (mm)	Acquisition time
3D FLAIR imaging	Sagittal	6,000/390 (TI 2,000)	–	256*184	0.57 (over contiguous)	4 min 42 s
3D double inversion recovery imaging	Sagittal	5,500/298 (TI 2,550/450)	–	208*163	0.65 (over contiguous)	5 min 13 s
Susceptibility weighted imaging	Transverse	31/7.2 (echo spacing 6.2, 4echo)	17	384*300	1.00 (over contiguous)	4 min 52 s
3D T1 weighted imaging	Sagittal	7.6/3.6	10	288*288	0.9	4 min 42 s
2D T2 weighted imaging	Transverse	7,000/90	–	384*345	3.0/0.5	2 min 43 s

### 2.4. SVD scores

The HA-SVD score was evaluated as follows; 1 point was given for the severity of each of the four MRI markers (lacunar infarcts, MBs, BG-PVS, and WMH), with a minimum score of 0 and a maximum score of 4 (Klarenbeek et al., 2013). The CAA-SVD score was calculated as sum of points for the severity of each of the four markers (lobar MBs, cSS, CSO-PVS, and WMH) with a minimum score of 0 and a maximum score of 6 (Charidimou et al., 2016). Specifically as follows: For lobar MBs, 1 point was given if two to four MBs were present, and 2 points were for five or more MBs. The presence of cSS was given 1 point if focal and 2 points if disseminated. The presence of CSO-PVSs was confirmed if there were moderate to severe (>20) PVSs (1 point if present). For WMH, 1 point was given if the score on the Fazekas scale was 3 for periventricular WMH and/or 2 or 3 for deep WMH. The modified CAA-SVD score was evaluated as follows (Matsuda et al., 2021): For posteriorly dominant WMH, tissue quantification was performed using in-house software (FUsed Software for Imaging Of Nervous system: FUSION) (Tabei et al., 2017). If there was a large amount of posterior WMH, 1 point was added to the CAA-SVD score. CMIs due to CAA were confirmed when CMIs localized within the cortex, predominantly in the occipital lobe, were smaller than 5 mm in diameter, and there were fewer than three lesions (Ishikawa et al., 2020). When there were any CMIs due to CAA, we added 1 point to the CAA-SVD score. Three raters (M.K, I.Y, and U.Y) independently assessed these scores.

### 2.5. Statistical analyses

Differences in demographic variables and results from the neuropsychological assessment between patients with unchanged CDR after 1 year (group A) and those with worsened (group B) were analyzed using a two-sample *t*-test for equal variance data, the Welch test for unequal variance data. In addition, regression analysis was performed using the significantly different items as covariates and the change in CDR as the objective variable.

Statistical analysis was performed using IBM SPSS Statistics software version 25 (IBM Corp., Armonk, NY, USA). Clinical and radiological characteristics were presented as numbers with percentages and means with standard deviation. Statistical analyses were performed using IBM SPSS Statistics software version 25 (IBM Corp., Armonk, NY, USA). Differences with *p* < 0.05 were considered statistically significant.

## 3. Results

### 3.1. Patients

In group A, the mean age was 71.8 years with a standard deviation (SD) of 2.4 years, the mean disease duration was 15.8 months with a SD of 4.9 months, and 12 patients (60%) met the modified Boston criteria for CAA (version 1.5) (Greenberg and Charidimou, 2018). Regarding vascular risk factors, nine patients had hypertension (45%) and five (25%) had dyslipidemia. The global CDR score was 0.5 for 15 patients (75%) and 1.0 for five patients (25%), with a mean CDR-sum of boxes scores of 3.1 with a SD of 0.3. Of the five patients with a global CDR score of 1.0, four were diagnosed with probable AD and one with vascular dementia by their respective criteria. Among the 15 patients with MCI, six had aMCI (40%), and nine had naMCI (60%). In group B, the mean age was 75.3 years with a SD of 2.1 years, the mean disease duration was 15.2 months with a SD of 5.6 months, and six patients (66.6%) met the modified Boston criteria for CAA (version 1.5). Regarding vascular risk factors, five patients had hypertension (55.5%) and one (11.1%) had dyslipidemia. The global CDR score was 0.5 for six patients (66.6%) and 1.0 for three patients (33.3%), with a mean CDR-sum of boxes scores of 4.3 with a SD of 0.7. All patients with a global CDR score of 1.0 met the criteria for probable AD. Among the six patients with MCI, five had aMCI (83.3%) and one had naMCI (16.6%). After 1 year, four of five patients with aMCI had converted to AD and one to vascular dementia; one patient with naMCI had converted to AD (Table 3).

**TABLE 3:** Diagnoses before and after aggravation in group B.

Patient	Diagnosis		MMSE		Lacune, No.		Lobar MBs, No.		Deep MBs, No.		WMH (Fazekas; deep), score		WMH (Fazekas; Periventricular), score		CSO-PVS, score		BG-PVS, score	
	Base-line	Follow-up	Base-line	Follow-up	Base-line	Follow-up	Base-line	Follow-up	Base-line	Follow-up	Base-line	Follow-up	Base-line	Follow-up	Base-line	Follow-up	Base-line	Follow-up
1	probable AD	probable AD	23	16	0	0	0	2	0	0	3	3	3	3	4	4	1	2
2	aMCI	probable AD	22	15	0	0	3	6	0	0	1	1	1	1	1	3	1	1
3	probable AD	probable AD	27	21	1	1	23	26	3	3	3	3	2	3	3	3	3	3
4	aMCI	probable AD	21	10	0	0	43	47	3	3	2	2	3	3	4	4	2	2
5	aMCI	vascular dementia	23	19	2	2	3	3	0	0	3	3	3	3	3	3	2	2
6	naMCI	probable AD	27	27	0	0	0	0	0	0	2	2	3	3	3	3	3	3
7	probable AD	probable AD	25	22	0	0	12	12	0	0	2	2	2	3	4	4	3	3
8	aMCI	probable AD	27	26	0	0	0	6	0	0	2	2	1	2	4	4	1	1
9	aMCI	probable AD	26	26	0	0	1	1	0	0	1	1	1	1	3	3	1	1

AD, Alzheimer’s disease; aMCI, amnestic mild cognitive impairment; naMCI, non-amnestic mild cognitive impairment; MMSE, Mini-Mental State Examination; MBs, microbleeds; WMH, white matter hyperintensities; CSO-PVSs, centrum semiovale enlargement of perivascular spaces; BG-PVSs, enlargement of perivascular spaces in the basal ganglia.

### 3.2. Longitudinal assessment of cognitive function

**FIGURE 2:**
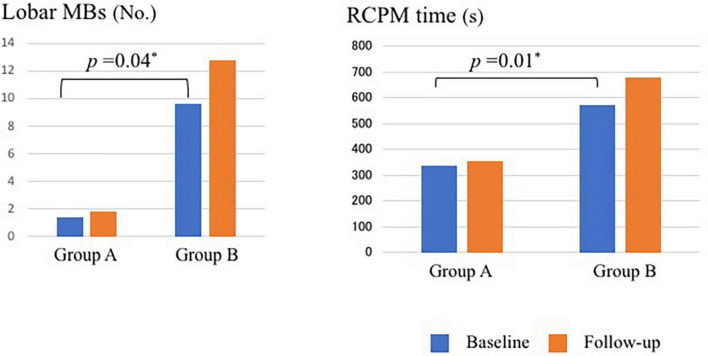
Number of MBs and time required for RCPM at baseline and follow-up in Group A and Group B. MBs, microbleeds; RCPM, Raven’s colored progressive matrices. *Differences with *p* < 0.05 was considered statistically significant.

Table 4 presents the longitudinal assessment of the participants’ cognitive function. At the baseline evaluation, the time required for RCPM (*p* = 0.01) (Figure 2) and TMT-A (*p* = 0.02) was significantly longer in group B. Group A had MMSE scores of 26.7 ± 0.5, RCPM scores of 26.9 ± 1.7, RBMT-SPS scores of 13.7 ± 1.4, and WF-letters recall of 6.4 ± 0.4 words. Group B had MMSE scores of 26.0 ± 0.9, RCPM scores of 25.0 ± 2.7, RBMT-SPS scores of 11.6 ± 3.0, and WF-letters recall of 5.6 ± 0.8 words. There was no significant difference in any of them. After 1 year, group A had MMSE scores of 25.4 ± 0.9, RCPM scores of 25.8 ± 1.4, RBMT-SPS scores of 15.3 ± 1.9, and WF-letters recall of 7.3 ± 0.6 words. Group B had MMSE scores of 20.1 ± 1.6, RCPM scores of 20.3 ± 2.0, RBMT-SPS scores of 7.2 ± 3.5, and WF-letters recall of 5.7 ± 2.0 words. Furthermore, group B had significantly lower MMSE (*p* = 0.03), RCPM (*p* = 0.02), and RBMT-SPS (*p* = 0.02) scores, and significantly fewer WF-letters recall (*p* = 0.03). After dividing the patients into two groups, one with markers and one without, and analyzing cognitive function for each marker, we found that the group with Lobar MBs had significantly less WF-letters recall (*p* = 0.014) and the group with Deep MBs had significantly longer time to TMT-A (*p* = 0.013). There were no significant differences in other imaging markers.

**TABLE 4:** Successive changes in the assessment of cognitive function.

Neuropsychological test		Initial evaluation			After 1 year evaluation		
		Group A, *N* = 20	Group B, *N* = 9	*p*	Group A, *N* = 20	Group B, *N* = 9	*p*
MMSE		26.7 (0.5)	26.0 (0.9)	0.30	25.4 (0.9)	20.1 (1.6)	0.03*
RCPM	Score	26.9 (1.7)	25.0 (2.7)	0.27	25.8 (1.4)	20.3 (2.0)	0.02*
	Time, s	334.7 (41.4)	573.5 (70.2)	0.01*	353.3 (26.5)	681.6 (194.6)	0.13
RBMT	SPS	13.7 (1.4)	11.6 (3.0)	0.29	15.3 (1.9)	7.2 (3.5)	0.02*
	SS	5.4 (0.8)	4.6 (1.5)	0.40	6.5 (1.0)	2.5 (1.5)	0.20
TMT	A, s	180.2 (19.4)	244.6 (48.9)	0.02*	164.9 (16.3)	256.5 (71.9)	0.03*
	B, s	221.2 (15.7)	294.8 (52.3)	0.19	226.4 (26.9)	343.7 (86.7)	0.09
WF,/min	Animal	13.4 (0.8)	12.6 (3.0)	0.33	14.2 (1.2)	11.7 (3.1)	0.08
	Letters	6.4 (0.4)	5.6 (0.8)	0.11	7.3 (0.6)	5.7 (2.0)	0.03*
MCAS	Score	3.1 (0.5)	3.0 (0.7)	0.60	2.6 (0.4)	2.0 (1.0)	0.91
	time, s	41.4 (7.4)	40.8 (9.8)	0.80	43.1 (7.4)	35.2 (14.9)	0.49

MMSE, Mini-Mental State Examination; RCPM, Raven’s Colored Progressive Matrices; s, seconds; RBMT, Rivermead Behavior Memory Test; SPS: Standard profile score, SS: screening score; TMT, Trail-Making Test; WF, word fluency; MCAS, Mie Constructional Ability Scale. *Differences with *p* < 0.05 was considered statistically significant.

For longitudinal changes in Groups A (Table 5) and B (Table 6), Group A had significantly less recall of WF-letters at follow-up compared to baseline (*p* = 0.04). And Group B had significantly lower (*p* < 0.01) MMSE scores at follow-up compared to baseline. Other items were also worsened, but not significantly.

**TABLE 5:** Successive changes in the assessment of cognitive function and image evaluation in group A.

Neuropsychological test		Baseline	Follow-up	*p*
MMSE		26.7 (0.5)	25.4 (0.9)	0.49
RCPM	Score	26.9 (1.7)	25.8 (1.4)	0.38
	Time, s	334.7 (41.4)	353.3 (26.5)	0.49
RBMT	SPS	13.7 (1.4)	15.3 (1.9)	0.45
	SS	5.4 (0.8)	6.5 (1.0)	0.36
TMT	A,s	180.2 (19.4)	164.9 (16.3)	0.56
	B,s	221.2 (15.7)	226.4 (26.9)	0.78
WF,/min	Animal	13.4 (0.8)	14.2 (1.2)	0.38
	Letters	6.4 (0.4)	7.3 (0.6)	0.04*
MCAS	Score	3.1 (0.5)	2.6 (0.4)	0.60
	time, s	41.4 (7.4)	43.1 (7.4)	0.86
**MRI findings**				
HA-SVD score, mean (SD)		1.8 (0.3)	2.0 (0.3)	0.02*
CAA-SVD score, mean (SD)		1.7 (0.3)	2.2 (0.3)	0.02*
modified CAA-SVD score, mean (SD)		2.0 (0.3)	2.8 (0.3)	<0.01*
MBs, No., mean (SD)	Deep	0.1 (0.1)	0.1 (0.1)	1.00
	Lobar	1.4 (1.4)	1.8 (0.4)	0.06
WMH (Fazekas), score, mean (SD)	deep	1.4 (0.1)	1.5 (0.1)	0.87
	Periventricular	1.4 (0.2)	1.7 (0.2)	0.78
Lacune, No., mean (SD)		0.3 (0.1)	0.3 (0.1)	1.00
CSO-PVS, score, mean (SD)		2.7 (0.2)	2.9 (0.2)	0.10
BG-PVS, score, mean (SD)		1.7 (0.2)	1.8 (0.2)	0.08
cSS, No., mean (SD)		0.2 (0.2)	0.3 (0.2)	0.85
posterior distribution of WMH, No. (%)		5 (25)	11 (55)	0.13
CMI due to CAA, No. (%)		0.0 (0.2)	0.0 (0.2)	1.00

MMSE, Mini-Mental State Examination; RCPM, Raven’s Colored Progressive Matrices; s, seconds; RBMT, Rivermead Behavior Memory Test; SPS, standard profile score, SS, screening score; TMT, Trail-Making Test; WF, word fluency; MCAS, Mie Constructional Ability Scale; MBs, microbleeds; WMH, white matter hyperintensities; CSO-PVSs, centrum semiovale enlargement of perivascular spaces; BG-PVSs, enlargement of perivascular spaces in the basal ganglia; cSS, cortical superficial siderosis; CMI, cortical microinfarcts.

*Differences with *p* < 0.05 was considered statistically significant.

**TABLE 6:** Successive changes in the assessment of cognitive function and image evaluation in group B.

Neuropsychological test		Baseline	Follow-up	*p*
MMSE		26.0 (0.9)	20.1 (1.6)	<0.01*
RCPM	Score	25.0 (2.7)	20.3 (2.0)	0.18
	Time, s	573.5 (70.2)	681.6 (194.6)	0.72
RBMT	SPS	11.6 (3.0)	7.2 (3.5)	0.18
	SS	4.6 (1.5)	2.5 (1.5)	0.65
TMT	A,s	244.6 (48.9)	256.5 (71.9)	0.72
	B,s	294.8 (52.3)	343.7 (86.7)	0.22
WF,/min	Animal	12.6 (3.0)	11.7 (3.1)	0.81
	Letters	5.6 (0.8)	5.7 (2.0)	0.86
MCAS	Score	3.0 (0.7)	2.0 (1.0)	0.56
	time, s	40.8 (9.8)	35.2 (14.9)	0.81
**MRI findings**				
HA-SVD score, mean (SD)		2.3 (1.2)	2.6 (0.5)	0.10
CAA-SVD score, mean (SD)		2.6 (0.6)	3.0 (0.5)	0.05
modified CAA-SVD score, mean (SD)		3.0 (0.5)	3.6 (0.5)	0.03*
MBs, No., mean (SD)	Deep	0.6 (0.4)	0.7 (0.4)	0.31
	Lobar	9.6 (4.8)	12.8 (5.6)	0.04*
WMH (Fazekas), score, mean (SD)	deep	2.2 (0.3)	2.2 (0.2)	0.89
	Periventricular	2.0 (0.4)	2.4 (0.2)	0.77
Lacune, No., mean (SD)		0.1 (0.1)	0.2 (0.2)	0.86
CSO-PVS, score, mean (SD)		3.6 (0.2)	3.6 (0.2)	1.00
BG-PVS, score, mean (SD)		1.4 (0.4)	1.6 (0.4)	0.31
cSS, No., mean (SD)		0	0	1.00
posterior distribution of WMH, No. (%)		1 (11.1)	4 (44.4)	0.23
CMI due to CAA, No. (%)		0	0	1.00

MMSE, Mini-Mental State Examination; RCPM, Raven’s Colored Progressive Matrices; s, seconds; RBMT, Rivermead Behavior Memory Test; SPS, standard profile score; SS, screening score; TMT, Trail-Making Test; WF, word fluency; MCAS, Mie Constructional Ability Scale; MBs, microbleeds; WMH, white matter hyperintensities; CSO-PVSs, centrum semiovale enlargement of perivascular spaces; BG-PVSs, enlargement of perivascular spaces in the basal ganglia; cSS, cortical superficial siderosis; CMI, cortical microinfarcts.

*Differences with *p* < 0.05 was considered statistically significant.

### 3.3. Changes of image findings and SVD scores over time

Table 7 shows the changes of image findings and SVD scores over time. At the baseline evaluation, group A had HA-SVD score of 1.8 ± 0.3, CAA-SVD score of 1.7 ± 0.3, and modified CAA-SVD score of 2.0 ± 0.3. The number of lobar MBs was 1.4 ± 1.4; the WMH had a Fazekas score of 1.4 ± 0.1 for deep and 1.4 ± 0.2 for periventricular, and 0.0 ± 0.2 CMI due to CAA, and five patients (25%) had posterior distribution of WMH. Group B had HA-SVD scores of 2.3 ± 1.2, CAA-SVD scores of 2.6 ± 0.6, and modified CAA-SVD scores of 3.0 ± 0.5. The number of lobar MBs was 9.6 ± 4.8; the WMH had a Fazekas score of 2.2 ± 0.3 for deep and 2.0 ± 0.4 for periventricular, and no CMI due to CAA, and one patient (11.1%) had a posterior distribution of WMH. Group B had significantly higher lobar MBs (*p* = 0.04) (Figure 2). Furthermore, group B had a higher Fazekas score of deep WMH (*p* = 0.06).

**TABLE 7:** Changes in image evaluation over time.

MRI findings		Baseline			Follow-up		
		Group A, *N* = 20	Group B, *N* = 9	*p*	Group A, *N* = 20	Group B, N = 9	*p*
HA-SVD score, mean (SD)		1.8 (0.3)	2.3 (1.2)	0.38	2.0 (0.3)	2.6 (0.5)	0.08
CAA-SVD score, mean (SD)		1.7 (0.3)	2.6 (0.6)	0.14	2.2 (0.3)	3.0 (0.5)	0.04*
modified CAA-SVD score, mean (SD)		2.0 (0.3)	3.0 (0.5)	0.14	2.8 (0.3)	3.6 (0.5)	0.03*
MBs, No., mean (SD)	Deep	0.1 (0.1)	0.6 (0.4)	0.44	0.1 (0.1)	0.7 (0.4)	0.40
	Lobar	1.4 (1.4)	9.6 (4.8)	0.04*	1.8 (0.4)	12.8 (5.6)	<0.01*
MBs, N (%)	Deep	1 (5)	2 (22.2)	0.36	1 (5)	3 (33.3)	0.11
	Lobar	12 (60)	6 (66.6)	0.89	12 (60)	8 (88.8)	0.32
WMH (Fazekas), score, mean (SD)	Deep	1.4 (0.1)	2.2 (0.3)	0.06	1.5 (0.1)	2.2 (0.2)	0.06
	Periventricular	1.4 (0.2)	2.0 (0.4)	0.19	1.7 (0.2)	2.4 (0.2)	0.12
Lacune, No., mean (SD)		0.3 (0.1)	0.1 (0.1)	0.87	0.3 (0.1)	0.2 (0.2)	0.98
Lacune, N, (%)		6 (30)	2 (22.2)	0.68	6 (30)	2 (22.2)	0.68
CSO-PVS, score, mean (SD)		2.7 (0.2)	3.6 (0.2)	0.36	2.9 (0.2)	3.6 (0.2)	0.28
BG-PVS, score, mean (SD)		1.7 (0.2)	1.4 (0.4)	0.76	1.8 (0.2)	1.6 (0.4)	0.86
cSS, No., mean (SD)		0.2 (0.2)	0	0.53	0.3 (0.2)	0	0.56
Posterior distribution of WMH, N, (%)		5 (25)	1 (11.1)	0.32	11 (55)	4 (44.4)	0.06
CMI due to CAA, No., (%)		0.0 (0.2)	0	0.85	0.0 (0.2)	0	0.85

MRI, magnetic resonance finding; HA-SVD, hypertensive arteriopathy small vessel disease; CAA-SVD, cerebral amyloid angiopathy small vessel disease; MBs, microbleeds; WMH, white matter hyperintensities; CSO-PVSs, centrum semiovale enlargement of perivascular spaces; BG-PVSs, enlargement of perivascular spaces in the basal ganglia; cSS, cortical superficial siderosis; CMI, cortical microinfarcts; SD, standard deviation. *Differences with *p* < 0.05 was considered statistically significant.

After 1 year, group A had HA-SVD scores of 2.0 ± 0.3, CAA-SVD scores of 2.2 ± 0.3, and modified CAA-SVD scores of 2.8 ± 0.3. The number of lobar MBs was 1.8 ± 0.4, the WMH had a Fazekas score of 1.5 ± 0.1 for deep and 1.7 ± 0.2 for periventricular, and 0.0 ± 0.2 CMI due to CAA, and 11 patients (55%) had a posterior distribution of WMH. Group B had HA-SVD scores of 2.6 ± 0.5, CAA-SVD scores of 3.0 ± 0.5, and modified CAA-SVD scores of 3.6 ± 0.5. The number of lobar MBs was 12.8 ± 5.6, the WMH had a Fazekas score of 2.2 ± 0.2 for deep and 2.4 ± 0.2 for periventricular, and no CMI due to CAA, and four patients (44.4%) had a posterior distribution of WMH. Group B had a significantly higher lobar MBs than group A (*p* < 0.01). Additionally, group B tended to have higher Fazekas score of deep WMH (*p* = 0.06). Furthermore, group B had significantly higher CAA-SVD (*p* = 0.04) and modified CAA-SVD scores (*p* = 0.03) than group A. Group B had higher HA-SVD score than group A, but the difference was not significant.

For longitudinal changes in Groups A (Table 5) and B (Table 6), HA-SVD scores, Group A had significantly increased scores (*p* = 0.023), while Group B had increased scores, but not significantly. CAA-SVD scores showed a significant increase (*p* = 0.02) for Group A and a significant trend (*p* = 0.059) for Group B. Modified CAA-SVD scores increased significantly for both Group A (*p* < 0.01) and Group B (*p* = 0.03).

### 3.4. Relationship between assessment of cognitive function and image evaluation

**FIGURE 3:**
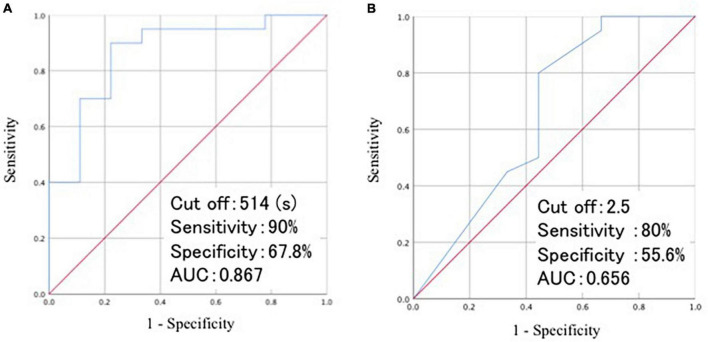
Visualization of ROC curves of the time required for RCPM **(A)** and the number of lobar MBs **(B)**. ROC, receiver operating characteristic; AUC, area under curve; RCPM, Raven’s colored progressive matrices; MB, microbleeds.

At baseline, the time required for RCPM and TMT-A, and the number of lobar MBs differed significantly between group A and B. We performed a regression analysis using these three variables as covariates and the change in CDR as the objective variable. The results showed that the time required for RCPM and the number of lobar MBs affected the change in CDR after 1 year (Table 8). Then we developed receiver operating characteristics curves for these two items (Figure 3). The time required for RCPM had a cutoff of 514 s, with a sensitivity of 90% and specificity of 67.8%. Furthermore, the number of lobar MBs had a cutoff of 2.5, with a sensitivity of 80% and specificity of 55.6%.

**TABLE 8:** Logistic regression analysis using change in CDR as the objective variable.

Variables	Coefficient (B)	Standard error	Wald χ 2	*p*	Odds ratio	95% CI for Exp
RCPM Time	0.011	0.005	5.737	0.001	1.011	1.002–1.020
Lobar MBs	0.470	0.227	4.278	0.000	1.599	1.025–2.496
TMT-A	–	–	–	0.466	–	–

CDR, clinical dementia rating; RCPM, Raven’s Colored Progressive Matrices; MBs, micobleeds; CI, confidence interval.

## 4. Discussion

Although several reports have examined factors that predict cognitive decline, and we thereby examined neuropsychological testing and MRI findings for predictive factors.

This study examined the prognostic value for the deterioration of cognitive functions and revealed the following findings. First, the delay in psychomotor speed from the time required for RCPM and TMT-A at baseline may predict cognitive deterioration. Second, the number of lobar MBs and the severity of deep WMH might predict cognitive decline. Finally, CAA-SVD and modified CAA-SVD scores could be associated with clinical symptoms.

At baseline, our results showed that the time required for RCPM and TMT-A was significantly longer in group B. This suggests that the RCPM and TMT-A time required may be a predictor of cognitive deterioration at 1 year. The time to perform RCPM and TMT-A is a measure of psychomotor speed, and previous reports have indicated that psychomotor speed is independently associated with the risk of incident AD and vascular dementia (Kuate-Tegueu et al., 2017). And low psychomotor speed has been associated with an increased risk of developing various brain outcomes, including dementia, AD, Parkinson’s disease, and depressive symptoms. Moreover, low psychomotor speed results from many individual brain abnormalities and can be a marker of brain vulnerability (Amieva et al., 2019). Indeed, in a study of MCI, deficits in verbal memory and psychomotor speed/executive functioning ability strongly predicted conversion to AD (Tabert et al., 2006). A study of aMCI also reported that the rate of change in TMT-B over 1 year was a predictor of conversion to AD (Rozzini et al., 2007). These studies were conducted in healthy elderly participants or MCI; however, the novelty of our study is that it suggested that psychomotor speed might also be a predictor of cognitive exacerbations in MCI and mild dementia cases.

In imaging, we found that group B had significantly higher number of lobar MBs and more severe deep WMH at baseline. There have been several reports on relationship between lobar MBs and cognitive dysfunction. A comparative study of AD patients with multiple MBs and those without MBs found that the group with multiple MBs had more severe cognitive impairment and more white matter hyperintensities (Goos et al., 2009). In a study with non-demented elderly patients with SVD, the presence and number of lobar MBs especially in the frontal and temporal lobes were related to global cognitive function, attention, and psychomotor speed (van Norden et al., 2011); A population-based cohort study of those without dementia showed that higher number of lobar MBs was associated with lower MMSE scores and psychomotor speed (Poels et al., 2012). In a memory clinic study of dementia, MCI, and those without cognitive impairment, the number of lobar MBs was predictor of cognitive worsening (Gyanwali et al., 2021). Our study, which included only MCI and mild dementia, similarly found that the number of lobar MBs is predictor of cognitive worsening. The amount of WMH significantly increases with increasing stages from healthy, MCI, mild AD, to moderate to severe AD (Kandiah et al., 2015). The severity and progression of WMH have been associated with a high risk of developing dementia (Brickman et al., 2015). Our study showed that deep WMH worsened at baseline in group B, and deep WMH may become a predictor of cognitive deterioration in MCI and mild dementia.

At baseline, the time required for RCPM and TMT-A and the number of lobar MBs differed significantly. We performed a regression analysis using these three items as covariates and the change in CDR as the objective variable. The results showed that the time required for RCPM and the number of lobar MBs affected the change in CDR after 1 year. TMT-A is generally presumed to be a test of visual search and psychomotor speed (Christopher and Harvey, 2006). In addition to psychomotor speed, the RCPM can assess several functions, including executive function, construct function, non-verbal reasoning, decision-making, and visuospatial ability (Sophie et al., 2006). Thus, the RCPM can assess more functions and may be more reflective of symptoms in patients with MCI and mild dementia than the TMT-A.

After 1 year, the CAA-SVD and the modified CAA-SVD scores were significantly higher in group B with worse CDR. This suggests that CAA and modified CAA-SVD scores were associated with worsening of clinical symptoms. In this study, six of nine patients (66.6%) in group B met the modified Boston criteria for CAA (version 1.5). Furthermore, among the six patients with MCI, five had aMCI (83.3%), and one had naMCI (16.6%). aMCI progresses at a higher rate to AD (Bennett et al., 2005).

Aging is one of the main causes of SVD; however, several other diseases, such as arteriosclerosis, CAA, genetic predispositions, and inflammation, cause SVD (Pantoni, 2010). In particular, hypertensive arteriosclerosis and CAA are the two major causes of SVD. In contrast, CAA is characterized by the progressive deposition of amyloid beta (Aβ) protein in the cerebral vessels; the major peptide isoforms of Aβ mainly consist of Aβ_1–40_ and Aβ_1–42_ (Pantoni, 2010). It is estimated that 80–100% of AD is complicated by CAA. In addition, continuums of vascular dementia and AD have been reported, and SVD has been associated with AD (Shindo et al., 2020). The involvement of vascular lesions in cognitive function of early AD is considered to be significant. The pathologies of SVD and AD coexist and synergistically worsen with advancing disease stages (Pfeifer et al., 2002). Patient characteristics may indicate that they have a pathological background of amyloid. CAA-SVD and modified CAA-SVD scores may reflect the pathological background of AD, which may explain our results. We previously reported that the CAA-SVD score may reflect cognitive function and also that the modified CAA-SVD score was significantly associated with cognitive function in patients visiting memory clinics (Matsuda et al., 2021). In the current study, although the CAA and modified CAA-SVD scores did not predict prognosis, these scores were associated with worsening of clinical symptoms. Moreover, our study showed that group B had a higher HA-SVD score at baseline. The HA-SVD score was mainly used for the evaluation of patients with lacunar stroke and vascular risk factors or either (Staals et al., 2014) and associated with intellectual functions (Huijts et al., 2013). Each SVD score has been associated with cognitive function; however, the longitudinal changes are still unclear.

This study has some limitations. First, it was based on a relatively small sample size. Therefore, type I error cannot be avoided. With a larger sample size, it may be possible to predict prognosis of clinical symptoms with CAA and modified CAA-SVD scores. Second, we were unable to conduct pathological examinations. These issues should be addressed in future studies. Despite these limitations, our study revealed that patients with MCI or mild dementia should be evaluated using CAA-SVD and the modified CAA-SVD scores. Furthermore, we suggested that patients with MCI and mild dementia with decreased psychomotor speed should be followed with caution.

In conclusion, this study revealed the imaging and neuropsychological predictor for cognitive deterioration in patients visiting memory clinics. After 1 year, patients with worsened CDR had a higher number of lobar MBs and prolonged psychomotor speed at baseline. These findings may contribute to the prevention of progression to dementia in patients visiting memory clinics.

## Data availability statement

The original contributions presented in this study are included in the article/Supplementary material, further inquiries can be directed to the corresponding authors.

## Ethics statement

The studies involving human participants were reviewed and approved by the Ethics Committee of Mie University Hospital. The patients/participants provided their written informed consent to participate in this study.

## Author contributions

KaM: draft of manuscript, acquisition of data, and analysis. K-iT and YI: revision of manuscript and interpretation of data. MSh, YU, NN, YH, HI, HM, KeM, MSa, MM, and RM: acquisition of data and interpretation of data. HT and AS: revision of manuscript, interpretation of data, and study supervision. All authors contributed to the article and approved the submitted version.
