# Supplementary material for: Magnetic resonance imaging and neuropsychological findings for predicting of cognitive deterioration in memory clinic patients
Source: Front Aging Neurosci. 2023 Aug 3;15:1155122. doi: 10.3389/fnagi.2023.1155122 (PMC10435295; doi:10.3389/fnagi.2023.1155122)
Supplement: Supplementary file 1 [file Table_1.DOCX]

supplementary table

We collected data from the patients who fulfilled the following inclusion criteria:

1) consulted with our memory clinic,

2) underwent neuroimaging examinations using 3T MRI,

3) completed neuropsychological assessments, and

4) had a global clinical dementia rating (CDR) score of 0.5 or 1.0 at enrollment.

Neuropsychological tests and CDR were performed within 3 months of MRI. No neurological events occurred between these tests and MRI. Exclusion criteria involved patients who

1) declined to or could not undergo MRI at 1 year,

2) declined neuropsychological and CDR assessments at 1 year, and

3) complications of other diseases.
